# Feasibility evaluation of novel AI‐based deep‐learning contouring algorithm for radiotherapy

**DOI:** 10.1002/acm2.14090

**Published:** 2023-07-18

**Authors:** Luis A. Maduro Bustos, Abhirup Sarkar, Laura A. Doyle, Kelly Andreou, Jodie Noonan, Diana Nurbagandova, SunJay A. Shah, Omoruyi Credit Irabor, Firas Mourtada

**Affiliations:** ^1^ Department of Radiation Oncology Christiana Care Helen F. Graham Cancer Center Newark Delaware USA; ^2^ Department of Radiation Oncology Thomas Jefferson University Hospital Philadelphia Pennsylvania USA

**Keywords:** artificial intelligence, contouring, convolutional neural networks, deep‐learning, segmentation

## Abstract

**Purpose:**

To evaluate the clinical feasibility of the Siemens Healthineers AI‐Rad Companion Organs RT VA30A (Organs‐RT) auto‐contouring algorithm for organs at risk (OARs) of the pelvis, thorax, and head and neck (H&N).

**Methods:**

Computed tomography (CT) datasets from 30 patients (10 pelvis, 10 thorax, and 10 H&N) were collected. Four sets of OARs were generated on each scan, one set by Organs‐RT and the others by three experienced users independently. A physician (expert) then evaluated each contour by assigning a score from the following scale: 1‐Must Redo, 2‐Major Edits, 3‐Minor Edits, 4‐Clinically usable. Using the highest‐scored OAR from the human users as a reference, the contours generated by Organs‐RT were evaluated via Dice Similarity Coefficient (DSC), Hausdorff Distance (HDD), Mean Distance to Agreement (mDTA), Volume comparison, and visual inspection. Additionally, each human user recorded the time to delineate each structure set and time‐saving efficiency was measured.

**Results:**

The average DSC obtained for the pelvic OARs ranged between (0.81 ± 0.06)_Rectum_ and (0.94 ± 0.03)_Bladder_. (0.75 ± 0.09)_Esophagus_ to ${\left(0.96\pm 0.02\right)}_{\mathrm{Rt}.\;\mathrm{Lung}}$ for the thoracic OARs and (0.66 ± 0.07)_Lips_ to (0.83 ± 0.04)_Brainstem_ for the H&N. The average HDD in cm for the pelvis cohort ranged between (0.95 ± 0.35)_Bladder_ to (3.62 ± 2.50)_Rectum_, (0.42 ± 0.06)_SpinalCord_ to (2.09 ± 2.00)_Esophagus_ for the thoracic set and ${\left(0.53\pm 0.22\right)}_{\mathrm{Cerv}\_\mathrm{SpinalCord}}$ to (1.50 ± 0.50)_Mandible_ for the H&N region. The time‐saving efficiency was 67% for H&N, 83% for pelvis, and 84% for thorax. 72.5%, 82%, and 50% of the pelvis, thorax, and H&N OARs were scored as clinically usable by the expert, respectively.

**Conclusions:**

The highest agreement registered between OARs generated by Organs‐RT and their respective references was for the bladder, heart, lungs, and femoral heads, with an overall DSC≥0.92. The poorest agreement was for the rectum, esophagus, and lips, with an overall DSC⩽0.81. Nonetheless, Organs‐RT serves as a reliable auto‐contouring tool by minimizing overall contouring time and increasing time‐saving efficiency in radiotherapy treatment planning.

## 1 INTRODUCTION

Contouring CT scans is an essential task in radiotherapy treatment planning. This allows for the optimization of the therapeutic ratio by spatially mapping out the patients’ anatomy and enabling quantitative assessments of dose distributions. Manual contouring is very time‐consuming, and it often leads to large interobserver variability as it is subjective to judgement of the user.^1^, ^2^ Jamenson et al.^3^ argued that these variations are the most significant contribution to uncertainties in radiotherapy treatment planning.

The ever‐increasing demand for accuracy and precision in radiotherapy, primarily due to the emergence of more complex treatment modalities, such as SRS and SBRT, exacerbates the clinical challenges posed by manual contouring. The development of atlas‐based auto‐contouring algorithms was the first attempt to address this issue. In the past 10 years, the use of atlas‐based solutions has become widespread, alongside the emergence of deep‐learning, AI‐based algorithms.^4^, ^5^ Recently, the latter began to show promising results over atlas‐based solutions.^6^

One of the limitations of atlas‐based auto‐contouring products is that their segmentation is based on a compilation of previously contoured CT data sets.^7^ One of these older CT scans is registered to the new scan and the contours transferred over. The accuracy in this process depends on differences in image quality between new and older CT scans, type of registration, post‐processing techniques, and so forth,^8^ Further, atlas‐based algorithms take up to several minutes of processing and use significant computing power.^9^ Deep‐learning algorithms, on the other hand, employ convolution neural networks also trained with a large number of contoured images but optimized to recognize spatial variations of specific objects in the image.^8^

AI‐Rad Companion Organs RT (Organs‐RT) is a recently developed and FDA‐approved cloud‐based auto‐contouring solution from Siemens Healthineers (Erlangen, Germany) that uses an AI‐based algorithm to delineate OARs. This solution uses an independently trained deep reinforcement learning technique to identify and distinguish anatomical landmarks within or near the OAR to be delineated.^10^, ^11^ The auto‐segmentation is then performed by a deep image‐to‐image network consisting of a convolutional encoder‐decoder architecture combined with a multi‐level feature concatenation.^11^, ^12^ The automatic segmentation is then carried out on a specific region of interest (ROI) focused on a single OAR at a time, thereby making it feasible for the algorithm to recognize anatomical variations of the organ.

This project aimed to assess the clinical feasibility of Organs‐RT by evaluating automatically generated OARs of the pelvis, thorax, and head and neck. The assessment includes a measure of time‐saving efficiency as compared to our current clinical workflow, which encompasses a combination of manual contouring and atlas‐based tools in RayStation V10.01 (Stockholm, Sweden). This study was approved by the institution review board (IRB).

## 2 METHODS AND MATERIALS

Thirty CT scans were selected from the Raystation (RS) treatment planning database. With the patients in the head‐first supine position, the scans consisted of 10 pelvis, 10 thorax, and 10 head and neck (H&N). Three experienced users, a medical physicist, and two dosimetrists, manually generated a set of contours each, using atlas‐based auto‐segmentation tools in RS. The libraries of the atlas‐based algorithm deployed contained at most 15 CT data sets each. The human‐delineated set (C_Manual_) for the pelvis and H&N followed the RTOG guidelines for the specific body sites.^13^, ^14^ The time to generate each C_Manual_ was recorded.

Per department standards, for conventional VMAT cases, the slice thickness for all scans was 3mm with a pitch of 0.6 for thorax and pelvis, and 0.8 for H&N. The pelvis cohort had all males, and the thorax cohort had all females. The H&N consisted of nine males and one female. One of the pelvises had a dual metal hip implant. Seven thoracic cases were acquired during Deep Inspiration Breath Hold (DIBH), and all H&N included a thermoplastic mask for setup reproducibility purposes. For the latter, seven patients bit onto a mouthpiece attached to the thermoplastic masks. The CT scans were sent to Organs‐RT and the time to receive the automatically generated set (C_Auto_) was recorded as t_Gen_. A radiation oncologist (expert) then evaluated each OAR and assigned a score from the following scale:1 – Must Redo, 2 – Major edits, 3 – Minor edits, 4 – Clinically usable.

To minimize human bias, the OARs were labeled in such a way as to make their source unidentifiable to the expert.

Dice Similarity Coefficient (DSC),^15^ Hausdorff distance (HDD),^16^ mean distance to agreement (mDTA), volume comparisons, and visual inspections were the metrics employed to evaluate C_Auto_. Provided that only three potential reference contours were generated per OAR, and the accuracy of consensus‐driven methods to merge contours, such as STAPLE, is highly dependent on the number of structures included,^17^ any of the human delineated OAR(s) scored as clinically usable was used as the ground truth for the comparisons. It should be noted that while only the OARs in C_Auto_ not scored as perfect were rectified, the metrics indicated above were performed on all of them. The DSC measures the overlap between two volumes, A and B, in the same CT scan.^18^ DSC=1 indicates perfect overlap, DSC=0 represents no overlap, and acceptable agreement is considered ≥0.7.^18^, ^19^ Mathematically, the DSC takes the form:

(1) $$\mathrm{DSC}\left(A,B\right)=\frac{2|A\cap B|}{\left|A|+|B\right|}$$

Where the expression |A∩B| represents the magnitude of the overlapping volume between surfaces A and B.

The HDD metric computes the largest separation between a point in set A to the closest point in set B in cm. This means that for perfect overlap, HDD=0. The calculation is carried out via the following maximum‐minimum function.^16^, ^20^

(2) $$\mathrm{HDD}\left(A,B\right)=\max\left\{{}_{a\in A}^{\mathrm{Sup}}\left[d\left(a,B\right)\right],\;{}_{b\in B}^{\mathrm{Sup}}\left[d\left(A,b\right)\right]\right\}$$

In this context, “a” and “b” represent the 3D location of a voxel on the surface of OARs “A” and “B,” respectively. The function $d\;\left(a,B\right)=\underset{a\in A}{\inf}d\left(a,b\right)\;$ establishes the distance between all voxels “a” and the closest voxel “b” while ${}_{a\in A}^{\mathrm{Sup}}\left[d(a,B)\right]$ selects the largest value in d(a, B). The symmetry in Equation (2) implies that ${}_{b\in B}^{\mathrm{Sup}}\left[d(A,b)\right]$ stores the longest separation between voxels on adjacent surface segments from OAR “B” to OAR “A.” Lastly, the operator max{}, returns the maximum voxel separation between the two OARs. The mDTA metric provides another geometrical comparison by computing the minimum distance between voxels on surfaces A and B but produces the mean or average distance between all points.^21^

The time efficiency was calculated by comparing the time spent, by the three humans, in contouring a given OAR set in all 10 cases (${\bar{t}}_{\mathrm{human}}$) to the time spent in generating them with Organs‐RT (t_OrgansRT_).

(3) $$t_{\mathrm{OrgansRT}}=P_{F}\;t_{\mathrm{Gen}}+F_{\mathrm{rect}}\left(t_{\mathrm{Gen}}+t_{\mathrm{rect}}\right)$$

The coefficients P_F_ and F_rect_ represent the fraction of C_Auto_ ranked as clinically usable without human intervention and the fraction to be rectified, respectively. t_Gen_ is Organs‐RT average time to generate the structures and t_rect_ is the time spent cleaning up the fraction assigned a score other than 4–Clinically usable.

(4) $$\mathrm{Efficiency}\;=\left(1-\frac{t_{\mathrm{OrgansRT}}}{{\bar{t}}_{\mathrm{human}}}\right)\;\times 100\%$$

## 3 RESULTS

**FIGURE 1:**
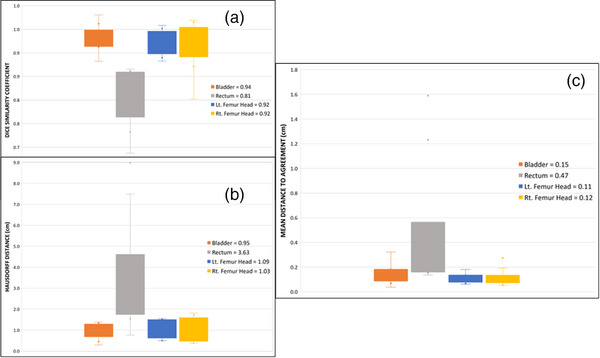
DSC (1a), HDD (1b), mDTA (1c) for pelvic OARs evaluated in this study. The metric averages for each are displayed on the right.

- Pelvis:

The expert ranking results for all OARs are outlined in Table 1. For the pelvis, Organs‐RT generated five bladders, seven rectums, nine left femoral heads, and eight right femoral heads with a clinically usable score of 4 without human intervention. Two femoral heads were scored as 1 – must redo. The results in Table 1 indicate that 72.5% of the OARs evaluated for the pelvis cohort were suitable for clinical use without human intervention while 22.5% required only minor adjustments. Figure 1 depicts the measured values for DSC, HDD, and mDTA for the pelvic OARs. The average value for each is also shown in the figure. In correlation to the results displayed in Table 1, the femoral heads and bladder showed the highest agreement between C_Auto_ and C_Manual_. The average DCS for these structures was >0.91 while that for the rectum was 0.81. A similar pattern was observed for HDD and mDTA values. The average HDD for bladder and femoral heads was <1.1 cm and for the rectum 3.63 cm. The mDTA averages for femoral heads and bladder fell <0.2 cm and the average for the rectum was 0.47 cm. Concerning the time‐saving efficiency recorded for the pelvic cases, an 83% increase in time efficiency was calculated using Equation 4. About 6.6 h were saved by sending the 10 pelvic cases to Organs‐RT for contouring.

$$t_{\mathrm{human}}=\;480\min,\;\;t_{\mathrm{OrgansRT}}=\;81.5\min\mathrm{Pelvis}$$

**TABLE 1:** Experts’ evaluation scores for OARs of the pelvis, thorax, and H&N generated by Organs‐RT.

	OAR	4–Clinically usable	3–Minor edit	2–Major edit	1–Must redo
Pelvis	Bladder	5	5	0	0
	Rectum	7	3	0	0
	Lt. Femoral head	9	0	0	1
	Rt. Femoral head	8	1	0	1
Thorax	Spinal cord	10	0	0	0
	Esophagus	6	2	1	1
	Heart	10	0	0	0
	Lt. Lung	7	3	0	0
	Rt. Lung	8	2	0	0
H&N	Brainstem	8	2	0	0
	Lips	2	8	0	0
	Mandible	2	8	0	0
	Spinal Cord	10	0	0	0
	Esophagus	3	6	1	0

**FIGURE 2:**
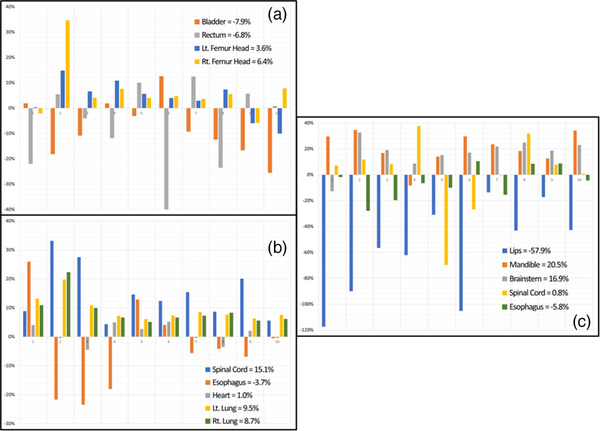
Volume differences registered between OARs generated by Organs‐RT and their respective ground truths for pelvis (a), thorax (b), and H&N (c).

Figure 2 illustrates the percent differences in volume for all evaluated OARs, and the corresponding average measurement for each. A negative average value indicates that the volume of C_Auto_was larger than that of C_Manual_, that is, when $\bar{V}<0\to C_{\mathrm{Auto}}>C_{\mathrm{Manual}}$ and vice versa. The rectum showed the largest differences in volume, though in 50% of the cases the C_Manual_ was larger. Organs‐RT slightly overestimated the volume of 70% of the bladders, and to an even lesser extend, it underestimated 80% of the left and right femoral heads.

**FIGURE 3:**
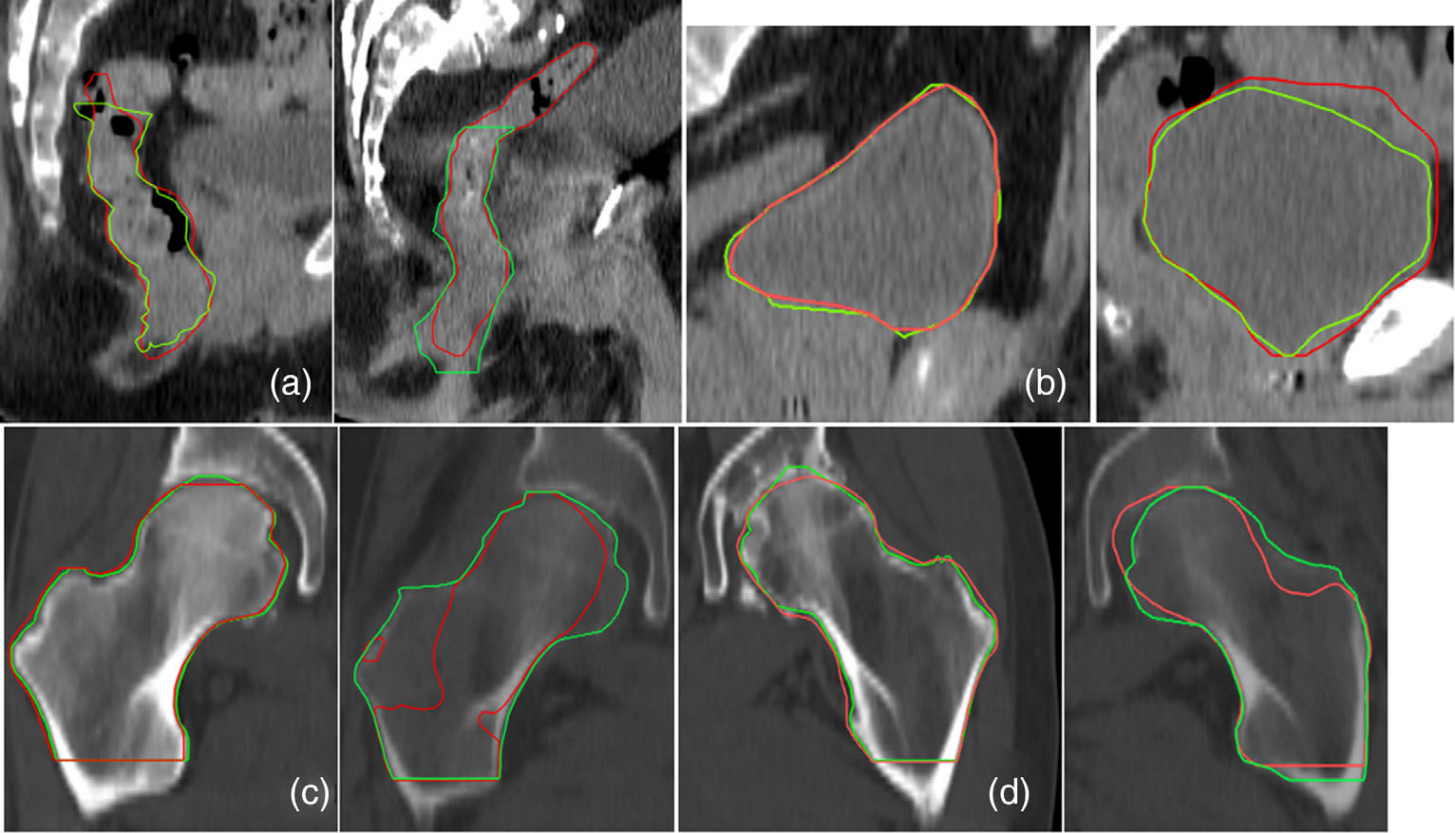
Highest (left) and lowest (right) agreement displays for the pelvic OARs evaluated in this study. The red are the contours generated by Organs‐RT while the green are the respective ground truths. Rectum (a), Bladder (b), Right Femoral Head (c), Left Femoral Head (d).

Figure 3 displays pairs of the pelvic OARs evaluated in this study. The left on each pair shows the OAR with the highest agreement to the ground truth, and the one on the right shows the highest discrepancy registered in the pelvis cohort. The window levels were set to facilitate visual inspection; for example, in Figure 3c,d, the femoral heads are shown in bone window level (WL) while the rectum and bladder (Figure 3a,b) are displayed in soft tissue WL. Note that these are 2D crosssections of each OAR on the same plane and the full volumetric discrepancy or agreement cannot be displayed. The rectums and bladders in the figure are displayed in the sagittal plane while the two femoral heads are shown in the coronal plane. By pairing OARs of highest and lowest discrepancy, these figures aim to illustrate the range of performance exhibited by Organs‐RT in this project.

- Thorax:

As displayed in Table 1, Organs‐RT was able to generate hearts and spinal cords (cord) with high enough accuracy for immediate clinical use. The left and right lungs were clinically usable about 75% of the time, with no significant edits. In the case of the esophagus, one was manually recontoured, three were cleaned up, and six were suitable for clinical use.

**FIGURE 4:**
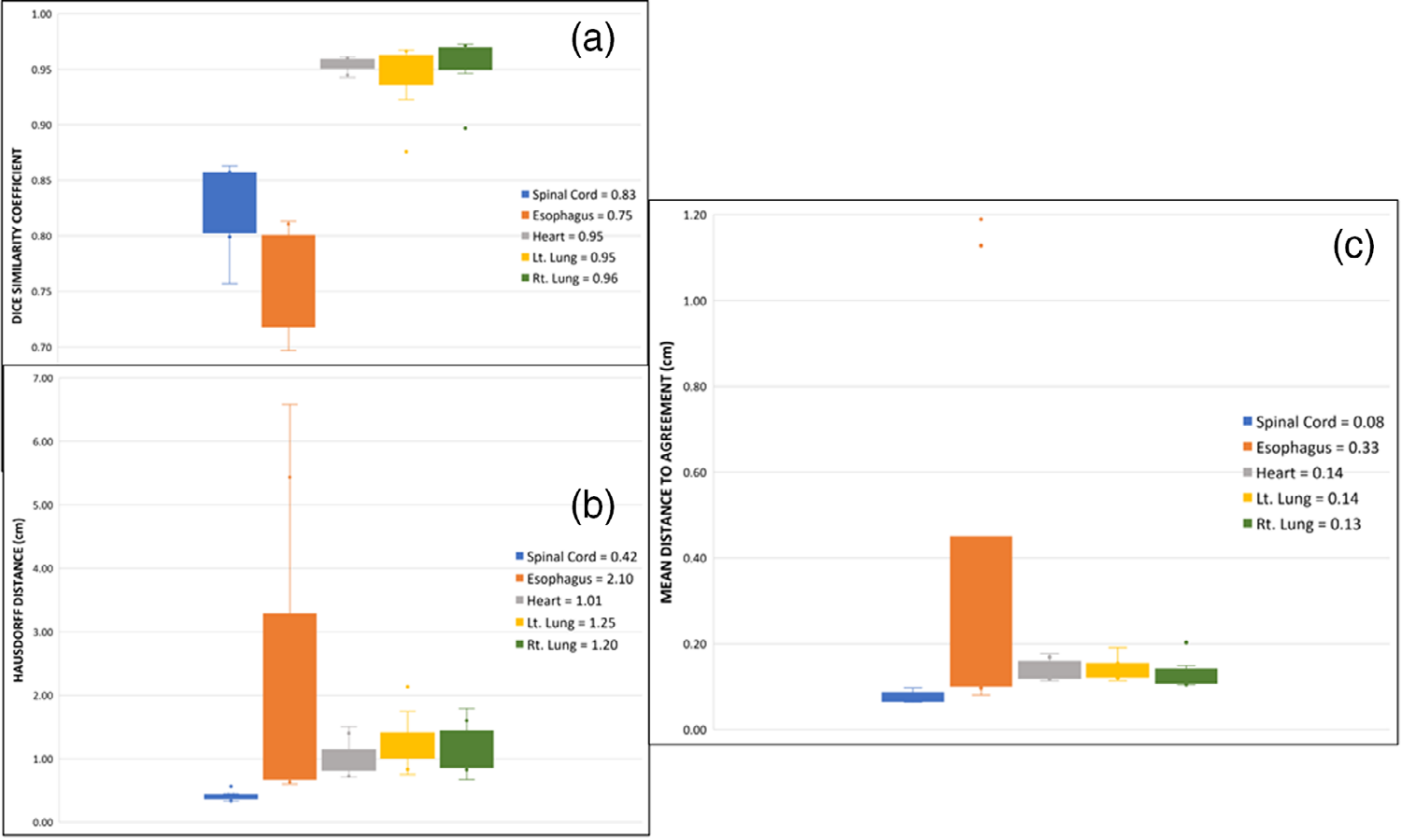
DSC (a), HDD (b), mDTA (c) for thoracic OARs evaluated in this study. The metric averages for each are displayed on the right.

The highest discrepancies were consistently observed for the esophagus. Figure 4 shows the average values for DSC, HDD, and mDTA of 0.75, 2.10, and 0.33 cm, respectively. For the other four structures, a mean DSC of 0.83 was obtained for the cord, even though all ten were optimal for clinical use without human intervention. The heart, left, and right lungs showed almost perfect scores with averages DSC>0.94, HDD<1.3 cm, and mDTA<0.2 cm. The distance comparisons for the cord were consistent, and not surprisingly, the shortest of all, HDD=0.42 cm and mDTA=0.08 cm. The time efficiency for the 10 thoracic cases was slightly higher than that of the pelvis cohort, 84%. The estimated time saved for all 10 cases was approximately 5.4 h.

$$t_{\mathrm{human}}=\;387.7\min,\;\;t_{\mathrm{OrgansRT}}=\;61.8\min\mathrm{Thorax}$$

In Figure 2b, the graph displays the percent differences in volume recorded for the thoracic cases. Although a few noticeable exceptions are observed, particularly in the first four cases, the average volume differences for the heart, left lung, and right lung remains within 10% of the ground truths. On average, the cord was 15.1% smaller than the ground truths, while the esophagus was larger around 60% of the time.

**FIGURE 5:**
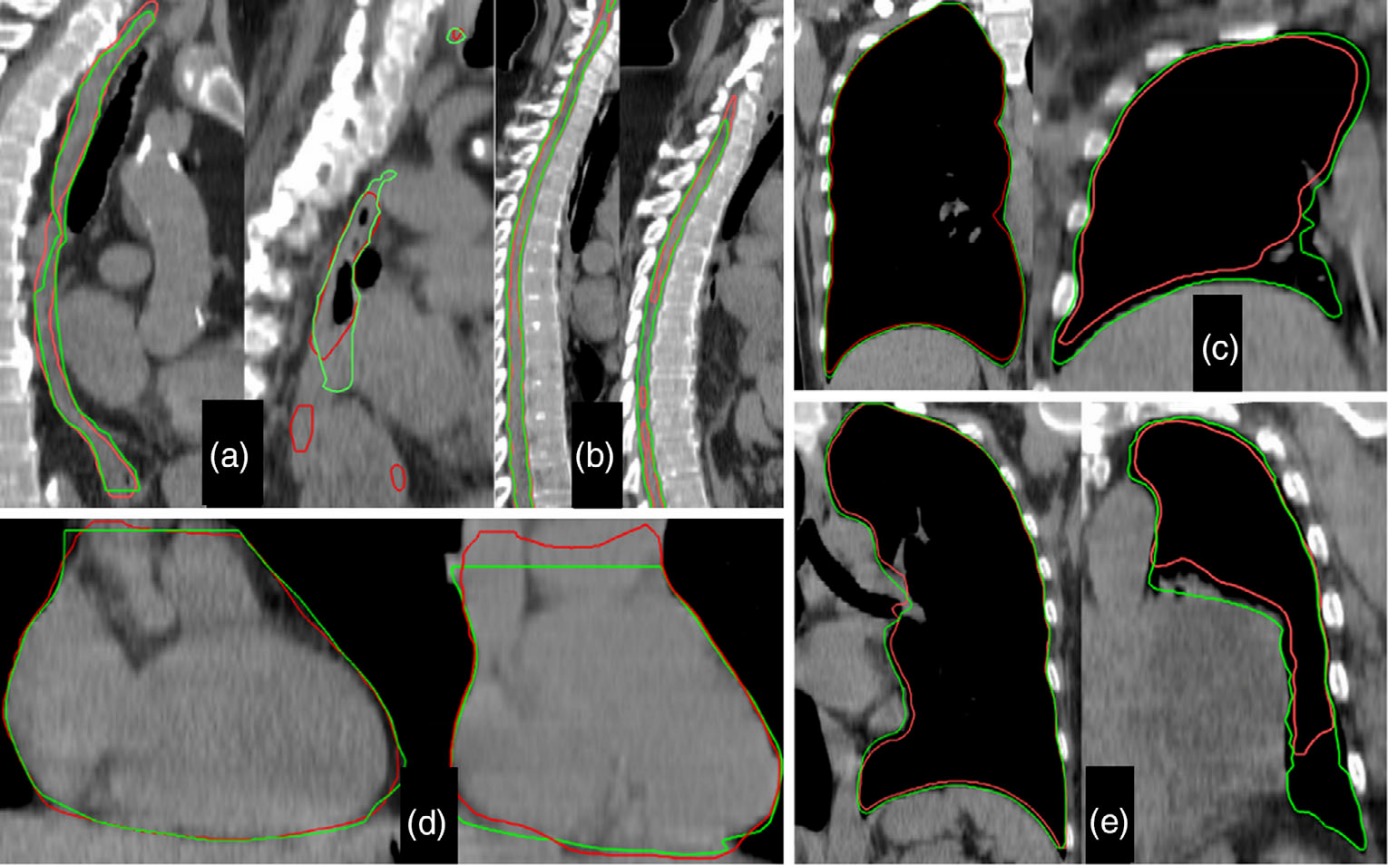
Highest (left) and lowest (right) agreement displays for the thoracic OARs evaluated in this study. The red are the contours generated by Organs‐RT while the green are the respective ground truths. Esophagus (a), Spinal Cord (b), Right Lung (c), Heart (d), Left Lung (e).

Similarly to Figure 3, Figure 5 depicts pairs of thoracic OARs with the highest and lowest agreement registered in the whole cohort. All displays are in soft tissue WL. The esophagus and cord (Figure 5a,b) are shown in the sagittal plane while the heart, left and right lungs (Figure 5c‐e) are shown in the coronal view.

- Head and Neck:

The expert's scores for the H&N cases are shown at the bottom of Table 1. The highest scores were assigned to the brainstems and cords as 80% and 100% of the cases were deemed satisfactory for clinical use without human intervention, respectively. The lips, mandible, and esophagus required minor adjustments the majority of the time. Similarly to the thorax cohort, only one esophagus showed the lowest overall score, in this case, requiring major edits. The two clinically usable lips belonged to the group of cases with no mouthpiece; it is believed that the mouthpieces played a role in the discrepancies between the C_auto_ and the the C_manual_; more on this in Section 4.

**FIGURE 6:**
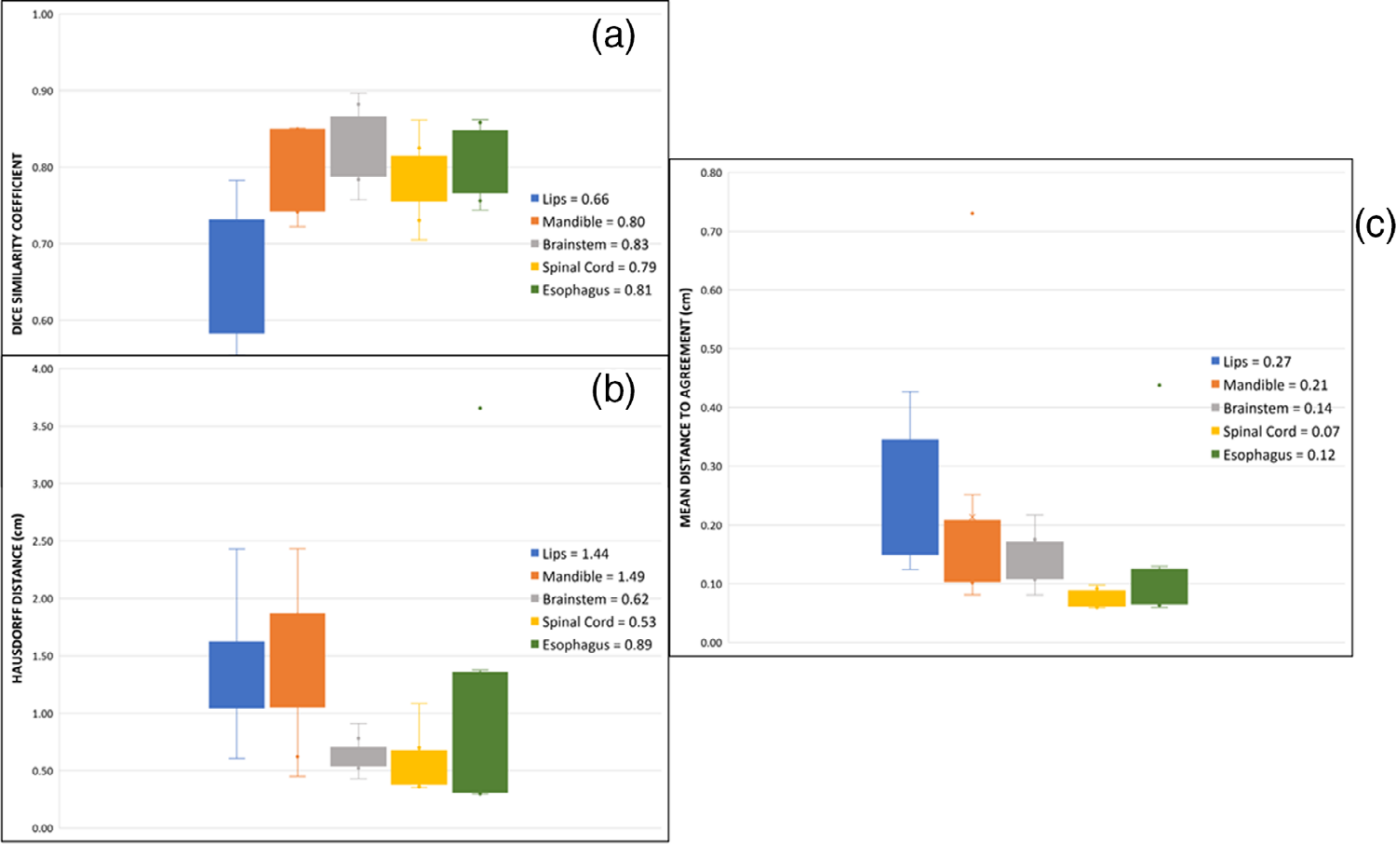
DSC (a), HDD (b), mDTA (c) for H&N OARs evaluated in this study. The metric averages for each are displayed on the right.

The DSC, HDD, and mDTA comparisons are displayed in Figure 6. Excluding the HDD, the poorest comparisons were consistently observed for the lips with average DSC=0.66, HDD=1.44 cm, and mDTA=0.27 cm. The largest HDD comparison belonged to the mandible with 1.49 cm. The average DSC for the mandible, brainstem, cord, and esophagus were very close to each other: mandible: 0.8, brainstem: 0.83, spinal cord: 0.79, and esophagus: 0.81. The HDD and mDTA showed significant agreement to ground truths for the brainstem and cord ${\mathrm{HD}\;D}_{\mathrm{Brainstem}}=0.62\;\mathrm{cm}$, ${\mathrm{HD}\;D}_{\mathrm{SpinalCord}}=0.53\;\mathrm{cm}$, ${\mathrm{mDT}\;A}_{\mathrm{Brainstem}}=0.14\;\mathrm{cm}$, and ${\mathrm{mDT}\;A}_{\mathrm{SpinalCord}}=0.07\;\mathrm{cm}$. On the other hand, the esophagus showed a HDD of almost 1 cm on average while the mDTA was only 0.12 cm. The time efficiency recorded for the H&N cases was 67%. This value is lower than that of the pelvis and thorax cases. This stems from an overall shorter human contouring time while having to clean up nine of the 10 cases. The average time saved for the H&N cohort was about 3.2 h.

$${\;t}_{\mathrm{human}}=\;286\;\min,t_{\mathrm{OrgansRT}}=\;93.3\;\min H\&N$$

The right of Figure 2 illustrates the volume comparison for the 10 H&N cases between C_Manual_ and C_Auto_. The most significant discrepancies registered corresponded to the lips. For these, 100% of the C_Auto_ were larger than C_Manual_. The only cases with no mouthpiece (5, 7, and 9 in Figure 2) showed better agreement between C_Auto_ and C_Manual_, even though C_Auto_ were still larger. In the case of the mandible and brainstem, C_Auto_were smaller than the ground truth 90% of the time. For the cord and esophagus, the differences in volume were 0.8% and −5.8%, respectively. This shows that for H&N OARs, excluding the lips, the volume differences fall within the range of the pelvis and thorax cohorts consistently.

**FIGURE 7:**
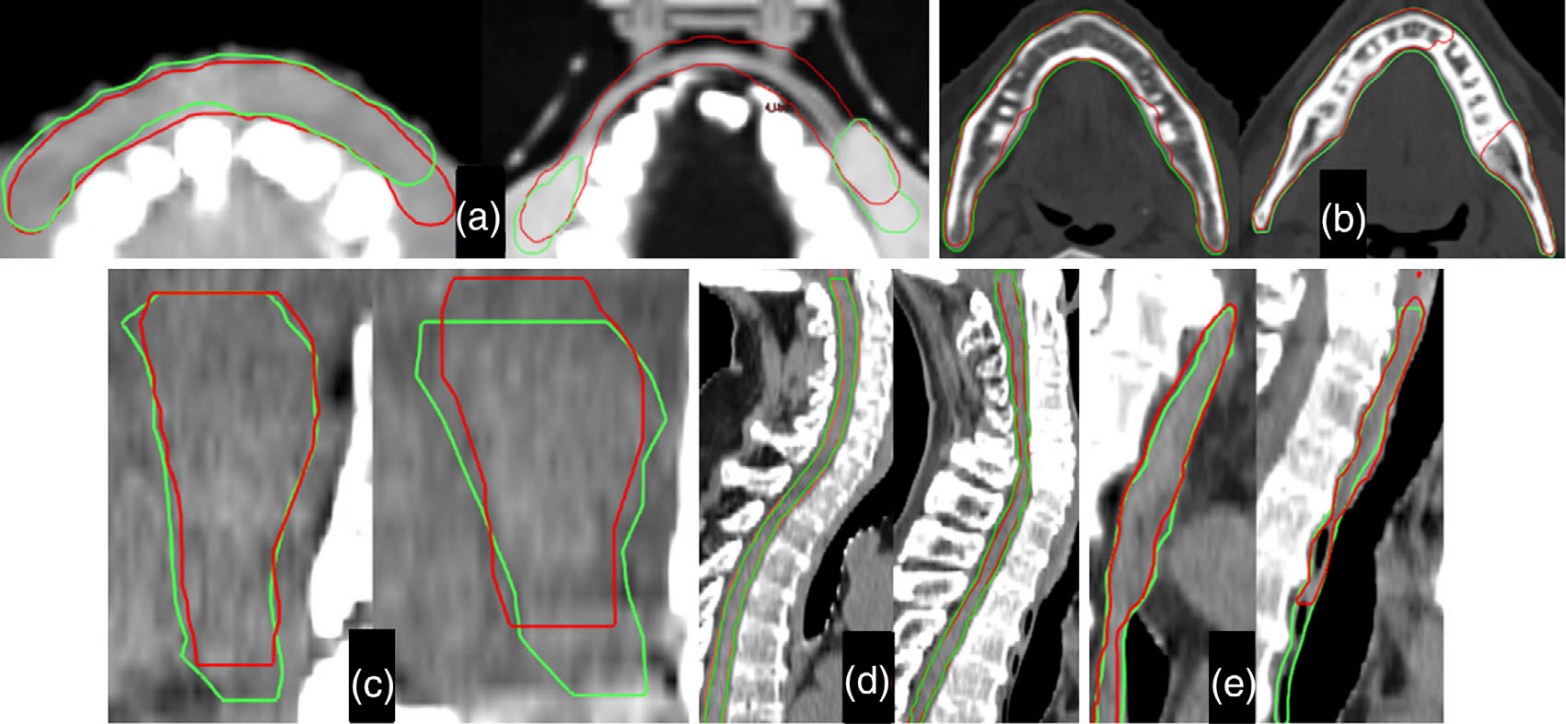
Highest (left) and lowest (right) agreement displays for the H&N OARs evaluated in this study. The red are the contours generated by Organs‐RT while the green are the respective ground truths. Lips (a), Mandible (b), Brainstem (c), Spinal Cord (d), Esophagus (e).

Figure 7 depicts pairs of the H&N OARs of highest and lowest agreement evaluated in this study. The brainstem, esophagus, and cord (Figure 7c–e) are shown in soft tissue WL while the mandibles (Figure 7b) are displayed in bone WL. The lips (Figure 7a) are shown in lung WL to display the mouthpieces. The mandibles and lips are displayed in the transversal plane while the others are shown in the sagittal view.

## 4 DISCUSSION

For all 30 cases, Organs‐RT successfully generated the OARs of interest without human intervention. While a few required manual adjustments, the net result of this study indicates that by incorporating Organs‐RT as a contouring tool prior to radiotherapy treatment planning, the time efficiency significantly increases. Given the recent FDA clearance for Organs‐RT for clinical use in November 2020, this project reveals valuable data not currently available. The most thorough quantitative assessment of Organs‐RT has been provided by Marschner et al.^11^— they evaluated Organs‐RT for pelvic and thoracic OARs with significantly larger population samples ($n_{\mathrm{pelvis}}=\;102$, $n_{\mathrm{thorax}}=\;237$). Despite the difference in the number of CT scans used in each study, the average DSC measured in both perfectly overlap. Our study also included the left femoral head, right femoral head, spinal cord, esophagus, lips, mandible, and brainstem, thus, providing a more comprehensive report for the functionality of Organs‐RT.

The rectum was one of the lowest‐performing OARs in this study. The results are in agreement with the studies by Bagalopal et al.^22^ (0.84 ± 0.03) and Rhee et al.^23^ (0.81), we measured 0.81 ± 0.06. Bagalopal et al. employed a deep‐learning algorithm with a 2D organ volume localization network before performing the 3D auto‐segmentation.^22^ Rhee et al. used 2464 CT scans to train an auto‐contouring algorithm based on a convolutional neural network (CNN).^23^ As mentioned earlier and also observed by Marschner et al.,^11^ the poor performance of Organs‐RT with rectum seems to be linked to the inclusion of the sigmoid as part of the rectum; the largest segment of sigmoid included in this study extends 8.9 cm into the sigmoid (Figure 1b and 3a, ). It should be noted that despite the persistent involvement of the sigmoid as part of the rectum, manual rectification is generally quick because the agreement in the lateral and anterior‐posterior directions of the actual rectum is often clinically acceptable. This can be observed in Figure 3a and inferred from the time‐saving efficiency recorded, 83%.

Due to their anatomy and location, the esophagus and cord were the only OARs evaluated in two cohorts, thorax and H&N. While the cord showed similar average scores in both groups, the esophagus resulted in higher discrepancies for the thoracic set. These differences may stem from two different factors. First, the scan lengths: the thoracic scans were longer than the H&N scans. On average, the thoracic scans’ superior end laid at the base of the skull (C1 vertebra) and extended inferiorly to L2/L3. The H&N scans included the whole brain and extended to the middle of the thoracic cavity (T6/T7). This means that in all H&N cases, only the cervical esophagus and cord were included and delineated. Meanwhile, the thorax cases comprised the entire esophagus and most of the cord.

Second, the surrounding tissue: independent of length, the cord is always surrounded by vertebral bodies. This tissue heterogeneity simplifies the localization of the structure and constraints any geometrical discrepancy to small and clinically acceptable magnitudes. The esophagus, on the other hand, is surrounded by soft tissue, especially inferior to the primary bronchi where the lungs gradually precede laterally away from it. Not surprisingly, the largest deviations were observed in this distal region. A small but commonly occurring deviation in the cervical esophagus occurred close to a vertebral body (usually C5); Organs‐RT often includes a region (a few mm deep) of the vertebral body as part of the esophagus. Additionally, some form of discontinuity was observed in four esophagi; three had one or a few slices missing (no more than 4)—axial slices with no esophagus delineated. The other contained a single transversal slice with two disjoint contours representing the esophagus (Figure 5a).

The average DSC for the esophagus (0.75±0.09) represents slightly higher agreement than observed by Yang et al.,^6^ at the AAPM Thoracic Auto‐Segmentation Challenge, where five of the seven algorithms involved used deep‐learning methods. Their average DSC ranged within 0.63±0.08.^6^ Zhu et al.^24^ evaluated interobserver variability by having three observers edit thoracic OARs generated by another CNN‐based auto‐contouring algorithm and comparing them to reference datasets. The average DSC scoring for the esophagus among the three observers varied between 0.74 and 0.81.^24^ As imperfect as Organs‐RT may be when delineating the esophagus, this high agreement in results attests to its potential as a reliable auto‐contouring tool as the OARs in the study by Zhu et al. were edited by experienced observers before evaluation.

The femoral heads showed strong consistency when compared to the reference OARs. As mentioned, one of the patients in the pelvis cohort underwent a dual metal hip implant. Although this case showed one of the lowest scorings, the most significant deviation was registered for a patient with osteoarthritis (Figure 3c,d). These results suggest that metal implants and the degradation of bone tissue can largely affect Organs‐RT's ability to effectively delineate femoral heads. In the assessment of AiContour (Linking MED), Chen et al.^25^ used manually delineated OARs by experienced physicians as a reference to evaluate those automatically generated by the AI‐based solution.^25^ Their average DSC for left and right femoral heads were 0.926 and 0.937, respectively. We obtained 0.92±0.04 for each. For bladder, this study measured an average DSC of 0.805 while we measured 0.94±0.03. These results render Organs‐RT as a superior tool over AiContour for pelvic OARs, not only for the higher agreement registered for the bladder but also for the similarities in scores for the femoral heads despite the inclusion of the two special cases mentioned above.

Similar to the results obtained by Marschner et al.,^11^ the heart and lungs were the OARs of the highest agreement to the ground truths. One of the free‐breathing FB thoracic scans showed the poorest, or second to poorest scoring in every metric used. Although it is intuitive to expect superior OAR‐delineating accuracy on DIBH thoracic CT scans considering the lack of patient motion during the acquisition, it might be too premature to draw any definitive conclusions relating to plausible dependencies of Organs‐RT on CT image quality between DIBH and FB scans.

The best‐performing OAR produced by Organs‐RT in the H&N cohort was the brainstem. The same results were observed in relatively similar studies by Van Dijk et al.^26^ and Urago et al.^27^ Each of their average DSC fell within 0.80 and 0.85, and we obtained 0.83±0.04. They compared brainstems generated by AI‐based solutions to sets manually delineated by expert users. Regarding volume, Organs‐RT slightly underestimated the dimensions of the H&N OARs, except for the lips (Figure 2). Similar findings were observed by Brouwer et al.^28^ In their study, they quantified how much manual editing was required for a series of H&N OARs generated by another deep‐learning algorithm. They also reported under‐segmentation by the AI‐based algorithm.^28^

The mandible required minor editing 80% of the time (Table 1). Notwithstanding, the average DSC of 0.80±0.04 is consistent with the value detected by Urago et al. ∼0.81.^27^ The mandibular region where most discrepancies were observed was the alveolar border. Eight mandibles showed noticeable inconsistencies in this region—generally less than 4 CT slices (Figure 7b). However, rectification was quick as it always consisted of removing excess segmentation from the center, in the transversal plane, and leaving only the mandibular rami demarcated.

As described earlier, seven H&N patients had a mouthpiece for positional reproducibility. This setup led to an inadequate assessment of Organs‐RT's ability to delineate the lips, as the mouthpieces were always included in the contours (Figure 7a). This can also be seen by the large discrepancies in volume (Figure 2), where the automatically generated lips were always larger than the ground truths. These differences in volume ranged from 42.7% to 117.4%, while for the cases with no mouthpieces (5, 7, 9 in Figure 2), the variation registered fell between 13.6% and 30.9%. Hence, displaying superior volumetric accuracy in the absence of mouthpieces.

This evaluation has a few limitations: The limited number of CT data sets in the atlas‐based algorithms deployed often produced inaccurate contours, resulting in longer manual contouring times. More sophisticated atlas‐based algorithms would minimize the time of human contouring and enable a more precise verification of time efficiency. The pelvis cohort contained only males, and the thorax only females. All cases had the patients in the head‐first supine position, and seven of the H&N patients had a mouthpiece. Due to anatomical differences between men and women, a study containing an equal number of males and females in different setup positions and without intrusive immobilization devices will extend the Organs‐RT's evaluated scope for clinical use. In addition, interobserver variability was not assessed in this study; this could impact the reliability of the manual contours used for comparison.

## 5 CONCLUSION

The clinical feasibility of Organs‐RT for OARs of the pelvis, thorax, and H&N was evaluated in this study. Organs‐RT delineated 140 OARs without human intervention. The metrics used demonstrated that Organs‐RT serves as a powerful auto‐contouring tool by significantly minimizing the manual contouring time and improving the efficiency of the radiotherapy treatment planning workflow.

## AUTHOR CONTRIBUTIONS

Firas Mourtada and Siemens Healthineers conceived the idea. Firas Mourtada, Kelly Andreou, and Siemens Healthineers developed the idea. Jodie Noonan, Diana Nurbagandova, and Luis A. Maduro Bustos provided the humanly‐delineated contours. SunJay A. Shah and Omoruyi Credit Irabor evaluated all the contours. Luis A. Maduro Bustos performed the computations and data analysis supervised by Firas Mourtada, Abhirup Sarkar, and Laura A. Doyle. Luis A. Maduro Bustos wrote the manuscript with revisions performed by Firas Mourtada, Siemens Healthineers, Jodie Noonan, Diana Nurbagandova, Laura A. Doyle, Abhirup, Sarkar, SunJay A. Shah, Omoruyi Credit Irabor, and Kelly Andreou.

## CONFLICT OF INTEREST STATEMENT

The authors declare no conflicts of interest.
